# Identification of two novel *MVD* mutations and one novel *FDPS* mutation in Chinese patients with porokeratosis

**DOI:** 10.3389/fmed.2026.1760287

**Published:** 2026-01-29

**Authors:** Yang He, Shengcai Zhu, Quan Wei, Fan Ye, Yunxia Zhu, Xiaoliang Ouyang, Liang Wu, Chunming Li

**Affiliations:** 1 Department of Dermatology, The Second Affiliated Hospital, Jiangxi Medical College, Nanchang University, Nanchang, Jiangxi, China; 2 Department of Plastic Surgery, The Second Affiliated Hospital, Jiangxi Medical College, Nanchang University, Nanchang, Jiangxi, China

**Keywords:** Chinese, *FDPS*, mutation analysis, *MVD*, porokeratosis

## Abstract

**Background:**

Porokeratosis (PK) is an autosomal dominant inherited disorder characterized by abnormal epidermal keratinization, primarily resulting from mutations in four genes associated with the mevalonate pathway: mevalonate decarboxylase (*MVD*), mevalonate kinase (*MVK*), phosphomevalonate kinase (*PMVK*), and farnesyl diphosphate synthase (*FDPS*).

**Purpose:**

The purpose of this study was to identify the causative mutations in seven sporadic cases of PK.

**Patients and methods:**

Clinical data and blood samples were collected from these seven sporadic cases. To identify pathogenic gene mutations in the patients, both whole-exome and Sanger sequencing were conducted. Bioinformatics resources such as PROVEAN, SIFT, PolyPhen-2, and Mutation Taster were employed to assess the pathogenicity of the identified mutations.

**Results:**

This study included seven sporadic cases of PK, comprising two cases of disseminated superficial actinic porokeratosis (DSAP), and five cases of disseminated superficial porokeratosis (DSP). We identified a total of five heterozygous mutations, including two novel *MVD* mutations (c.1122 + 1G > A, c.576G > T), one novel *FDPS* mutation (c.986A > C), and two *MVD* mutations that have been reported previously (c.1A > G and c.746 T > C). In conjunction with our previous study, we identified a total of six patients with the *MVD* c.746 T > C mutation from Jiangxi province, China, representing 50% of the total *MVD* mutation cases (12 in total).

**Conclusion:**

This research has expanded the database of mevalonate pathway genes associated with PK, thereby improving our comprehension of the fundamental mechanisms involved.

## 1 Introduction

Porokeratosis (PK) is an autosomal dominant inherited disorder characterized by epidermal keratinization. Clinically, it presents as one or more annular skin lesions, which exhibit central atrophy and depression, accompanied by a keratinized border. Histopathologically, it is defined by the presence of a keratin-like layer (1). According to clinical features, PK can be classified into six relatively prevalent subtypes: disseminated superficial actinic porokeratosis (DSAP), disseminated superficial porokeratosis (DSP), porokeratosis of Mibelli (PM), punctate porokeratosis (PP), porokeratosis palmaris et plantaris disseminata (PPPD), and linear porokeratosis (LP) (1, 2). PK is recognized as a genetically heterogeneous disorder, primarily resulting from mutations in one of four mevalonate pathway genes: *MVK*, *MVD*, *FDPS* and *PMVK* (3). Additionally, mutations in the *SLC17A9*, *FDFT1*, *SSH1*, and *SART3* genes may also be implicated as causative factors in PK (4–7).

In our earlier research, we discovered a new mutation in *MVD* (c.683G > C), a new *FDPS* mutation (c.438 T > G), along with three previously documented *MVD* mutations (c.1111_1113del, c.875A > G, and c.746 T > C) among PK cases from Jiangxi province in China (8). To gain deeper insight into the mutation spectrum associated with PK, we conducted a mutation analysis on genes in seven sporadic PK cases.

## 2 Materials and methods

### 2.1 Patients recruitment and sample collection

This study included seven sporadic cases of PK from Jiangxi province in China. Clinical data, along with around 5 mL of peripheral blood, were gathered from participants who signed informed consent. All the clinical diagnosis based on the manifestations and histopathological examinations. This study was approved by the Clinical Research Ethics Committee of the Second Affiliated Hospital of Nanchang University.

### 2.2 DNA extraction and whole-exome sequencing (WES)

Genomic DNA was extracted from peripheral blood samples using the Puregene Blood Core Kit B (QIAGEN, Hilden, Germany) in accordance with standard protocols. WES was carried out based on previously outlined methods (9). For each individual, 1.5 μg of genomic DNA was employed to create a captured library, which was later sequenced on the HiSeq X 10 platform (Illumina, San Diego, USA), yielding 150 bp paired-end reads. The raw data, totaling approximately 10 GB per exome, were aligned to the human reference genome sequence (GRCh38/hg38) employing the Burrows-Wheeler Alignment (BWA) tool.

### 2.3 Variant calling and gene filtration

In our study, we performed variant calling using the Genome Analysis Toolkit (GATK). Subsequently, we utilized ANNOVAR software for the comprehensive annotation of all identified variants. Our research specifically focused on identifying novel and rare protein-altering variants, which included various types of mutations such as frameshift, missense, nonsense, and critical splicing-site variations. We analyzed the frequency of these variants across different ethnic subgroups, leveraging data sourced from reputable genomic databases, including the 1,000 Genomes Project, the Exome Aggregation Consortium (ExAC), and the Genome Aggregation Database (gnomAD). Our primary focus was on alterations occurring within coding sequences, also known as exonic variants. Additionally, we investigated certain noncoding sequence variants located at the boundaries of exons and introns, which either had an unknown frequency or exhibited a minor allele frequency of less than 1%, as documented in the aforementioned databases.

### 2.4 Sanger sequencing

The design of primer pairs was accomplished using Primer 5.0 software to amplify the exons along with their respective exon/intron boundaries. The appropriate primers used for PCR amplifications included: *MVD* c.746 T > C (Forward Primer: TGTAAAACGACGGCCAGTAGGAGATGGCAT TGAGGTAAGAGATG; Reverse Primer: CAGGAAACAGCTATGACCGCAGGAGCCAAATG CAGAGAT), *MVD* c.1122 + 1G > A (Forward Primer: TGTAAAACGACGGCCAGTCAAATGAATGGACAAAGGCGTTCT; Reverse Primer: CAGGAAACAGCTATGACCGCTTAGAGAAACGGATGCATTCAC), and *FDPS* c.986A > C (Forward Primer: CAGAGAACAGGCACCAG CTTCA; Reverse Primer: GCTCTATCCCTTTCCACCAACTCA). Details of the PCR conditions can be provided upon request. The purification of PCR products was carried out utilizing SAP (Promega, Madison, WI, USA) in combination with Exo I (Epicentre, Madison, WI, USA). Sequencing of the purified PCR products was performed with the dye terminator chemistry BigDye3.1 (Applied Biosystems, Foster City, CA, USA). The sequencing reactions were analyzed on a 3730xl genetic analyzer from Applied Biosystems. For comparisons and analysis of the sequences, PolyPhred Analysis Software was employed.

## 3 Results

### 3.1 Clinical manifestations

**Figure 1:**
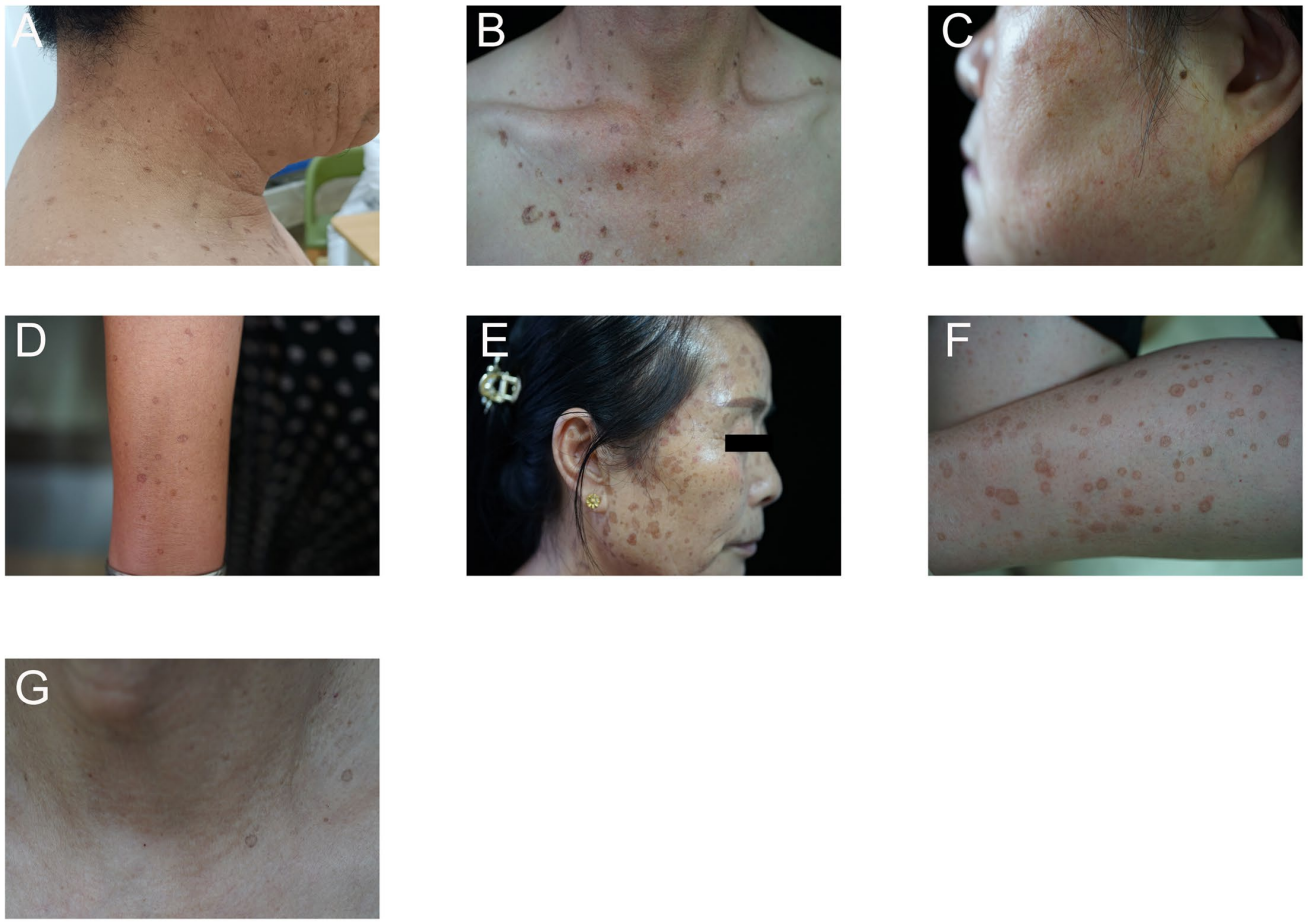
Clinical manifestations of seven sporadic cases of PK. **(A)** Sporadic case 1 (DSP); **(B)** Sporadic case 2 (DSP); **(C)** Sporadic case 3 (DSAP); **(D)** Sporadic case 4 (DSP); **(E)** Sporadic case 5 (DSP); **(F)** Sporadic case 6 (DSAP); **(G)** Sporadic case 7 (DSP).

This study comprised a total of seven sporadic cases of PK (Table 1). The first case involved a 62-year-old male who began to notice the development of multiple keratotic papules on his face, trunk, and limbs at the age of 52, as depicted in Figure 1A. The second case highlighted a 55-year-old male who exhibited typical keratotic papules on the trunk, arms, and legs since the age of 50, illustrated in Figure 1B. In the third case, a 54-year-old female presented with numerous keratotic lesions primarily located on areas of her body that were frequently exposed to the sun. These lesions had been persistent for 7 years, demonstrating a tendency to worsen during the summer months while showing signs of involution during the wintertime, as shown in Figure 1C. This patient had previously attempted to treat her condition with tretinoin ointment, though unfortunately, her symptoms did not improve. The fourth sporadic case featured a 52-year-old female who had been experiencing multiple small hyperkeratotic plaques for over a decade, as depicted in Figure 1D. The fifth case involved a 63-year-old female who developed multiple rounded hyperkeratotic plaques that displayed central atrophy and peripheral ridging, as illustrated in Figure 1E. The sixth case was a 70-year-old female who displayed a manifestation mirroring that of the third sporadic case, highlighted in Figure 1F. Lastly, the seventh case presented a 68-year-old male who developed small, scattered dark brown patches across his trunk, arms, and legs over a period of 6 months, as shown in Figure 1G. Sporadic cases 1, 2, 4, 5, and 7 were diagnosed with DSP, while sporadic cases 3 and 6 were diagnosed with DSAP.

**Table 1:** Summary of the clinical manifestations and mutations of 7 patients with PK.

Patient	Phenotype	Gender/age	Age at onset	Place of residence	Affected skin lesions			Gene	Mutation
					Face-neck	Trunk	Limbs		
Sporadic case 1	DSP	Male/62 y	52 y	Nanchang city	+	+	+	*MVD*	c.746 T > C
Sporadic case2	DSP	Male/55 y	50 y	Fengcheng city	−	+	+	*MVD*	c.1122 + 1G > A
Sporadic case3	DSAP	Female/54 y	47 y	Nanchang city	+	−	+	*MVD*	c.746 T > C
Sporadic case4	DSP	Female/52 y	40 y	Yongfeng country	−	+	+	*FDPS*	c.986A > C
Sporadic case5	DSP	Female/63 y	46 y	Nanchang city	+	+	+	*MVD*	c.1A > G
Sporadic case6	DSAP	Female/70 y	67 y	Nanchang city	+	−	+	*MVD*	c.746 T > C
Sporadic case7	DSP	Male/68 y	67 y	Xingguo country	−	+	+	*MVD*	c.576G > T

DSP, disseminated superficial porokeratosis; DSAP, disseminated superficial actinic porokeratosis.

### 3.2 Mutation analysis

**Figure 2:**
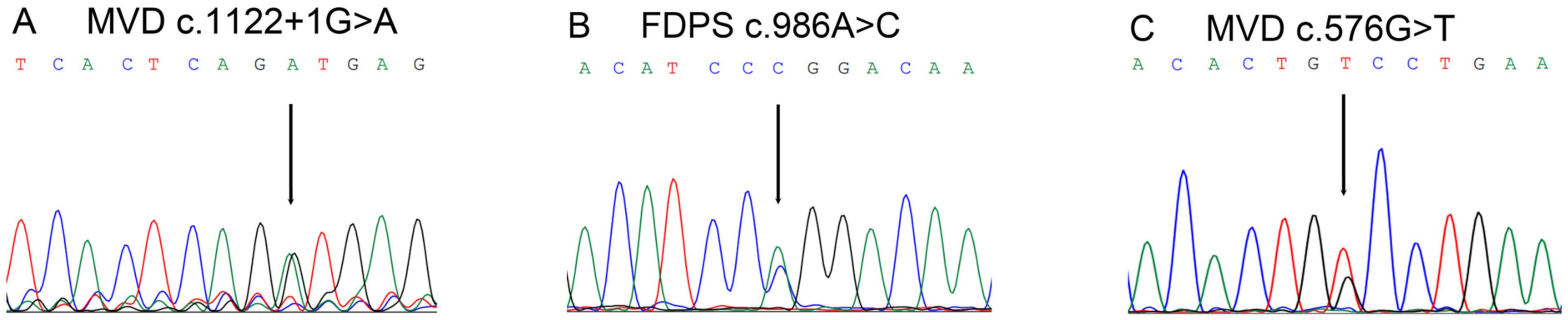
Genetic mutation of mevalonate pathway genes in this study. **(A)** A novel mutation *MVD* c.1122 + 1G > A was found in sporadic case 2. **(B)** A novel mutation *FDPS* c.986A > C (p. Q329P) was detected in sporadic case 4. **(C)** A novel mutation *MVD* c.576G > T (p. W192C) was identified in sporadic case 7. The black arrow indicates the mutation site.

Three novel mutations were identified among seven sporadic patients (Table 1). The novel mutation *MVD* c.1122 + 1G > A was identified in sporadic case 2 (Figure 2A). The novel mutation *FDPS* c.986A > C (p. Q329P) was detected in sporadic case 4; this missense mutation resulted in the substitution of glutamine with proline at codon 329 (Figure 2B). Additionally, the novel mutation *MVD* c.576G > T (p. W192C) was identified in sporadic case 7, leading to a substitution of tryptophan with cysteine at codon 192 (Figure 2C). Furthermore, the recurrent *MVD* mutation c.746 T > C (p. F249S) was observed in sporadic cases 1, 3, and 6. Another recurrent *MVD* mutation, c.1A > G (p. M1V), was found in sporadic case 5.

### 3.3 Bioinformatics analysis of the mutation

**Figure 3:**
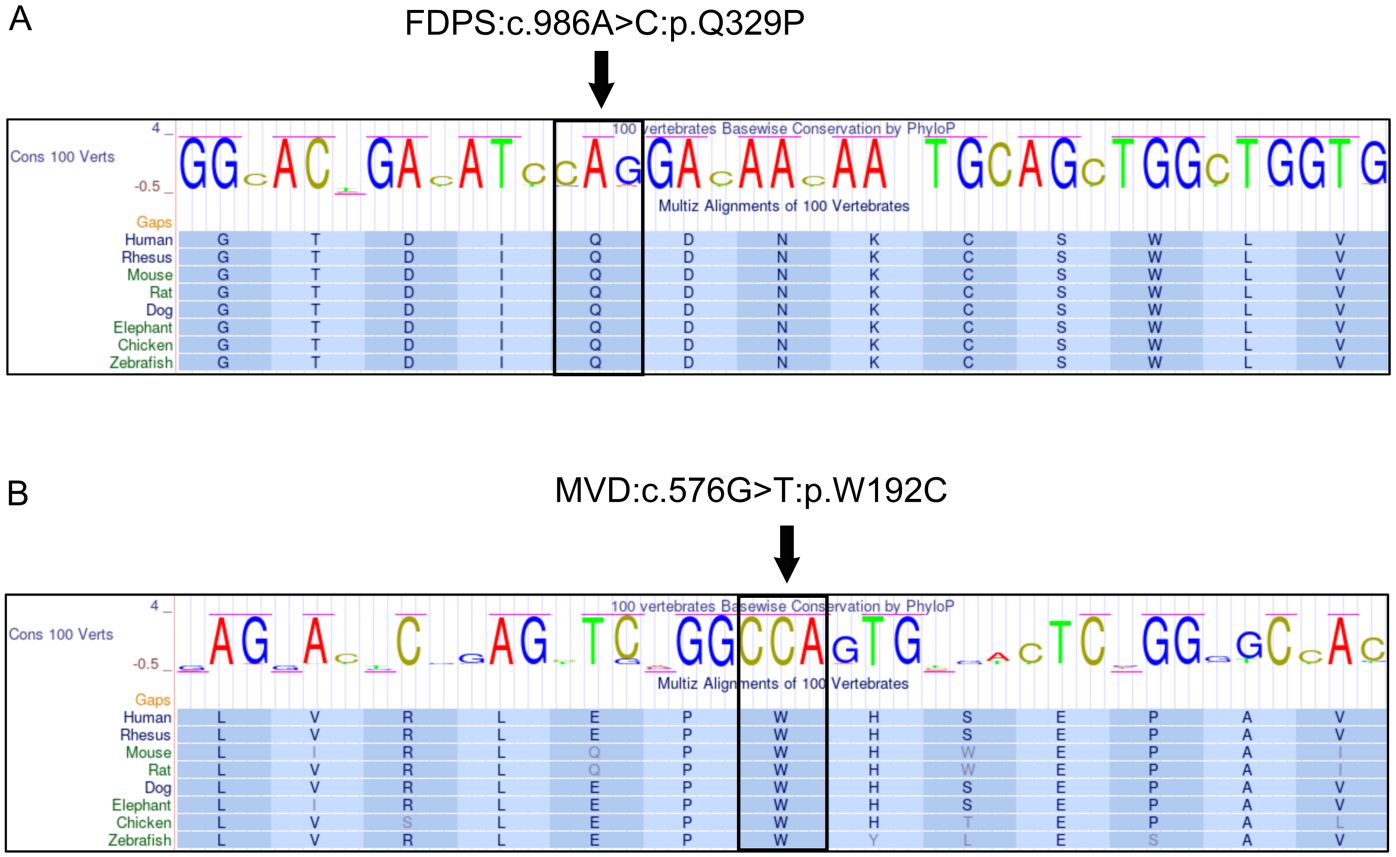
Conservation of amino acid sequences in the corresponding mutation of *FDPS* and *MVD* between species. The black arrow represents the amino acid at the mutated site. **(A)** c.986A > C (p. Q329P) mutation in *FDPS*. **(B)** c.576G > T(p. W192C) mutations in *MVD*.

The UCSC Genome Browser^1^ was utilized to investigate the evolutionary conservation of amino acids that have undergone mutations. An alignment of multiple sequences of homologous proteins from humans and various other vertebrates revealed that *FDPS* c.986A > C and *MVD* c.576G > T mutation occurred at highly conserved sites (Figure 3).

**Figure 4:**
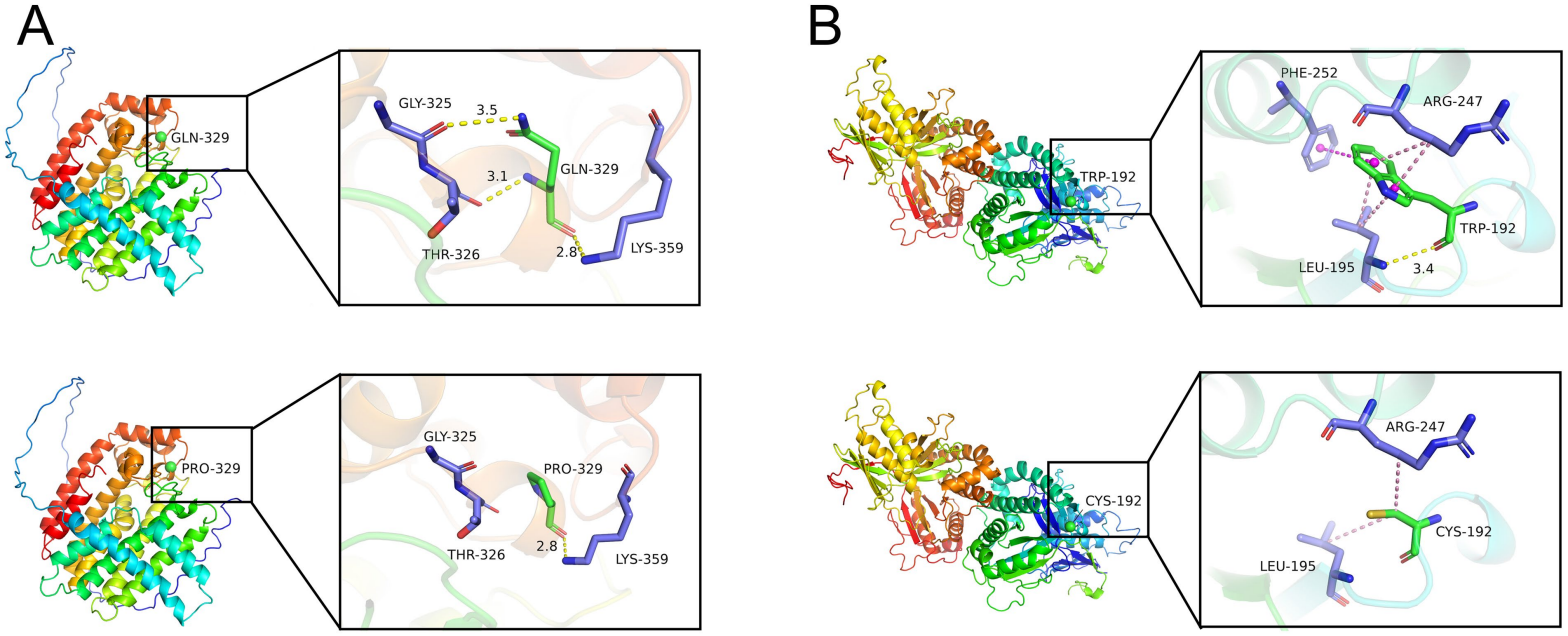
Three-dimensional structure of wild-type (WT) proteins and the novel mutant proteins. **(A)** The p. GLN329PRO mutation substitutes GLN329 with a non-polar proline, thereby abolishing the hydrogen bonds with GLY325 and THR326. **(B)** In the p. TRP192CYS mutant protein, the side chain of cysteine (CYS192) engages in weak alkyl interactions with ARG247 and LEU195, resulting in the complete abrogation of the hydrogen bond formed by WT TRP192.

To further explore the molecular structures of the wild type (WT) and mutant proteins, we constructed three-dimensional computer models using the I-TASSER server.^2^ As illustrated in Figure 4A, the WT protein contains a polar glutamine residue at position 329, which forms stable hydrogen bonds with three neighboring residues: GLY325, THR326, and LYS359. In contrast, the p. GLN329PRO mutation substitutes GLN329 with a non-polar proline, thereby abolishing the hydrogen bonds with GLY325 and THR326. As depicted in Figure 4B, the WT protein features a non-polar tryptophan at position 192 that forms a hydrogen bond with the adjacent leucine (LEU195). Furthermore, the aromatic indole ring of TRP192 engages in *π*-π stacking with the benzene ring of phenylalanine (PHE252) and forms π-alkyl interactions with the side chains of arginine (ARG247) and LEU195. In the p. TRP192CYS mutant protein, the side chain of cysteine (CYS192) engages in weak alkyl interactions with ARG247 and LEU195, resulting in the complete abrogation of the hydrogen bond formed by WT TRP192.

**Figure 5:**
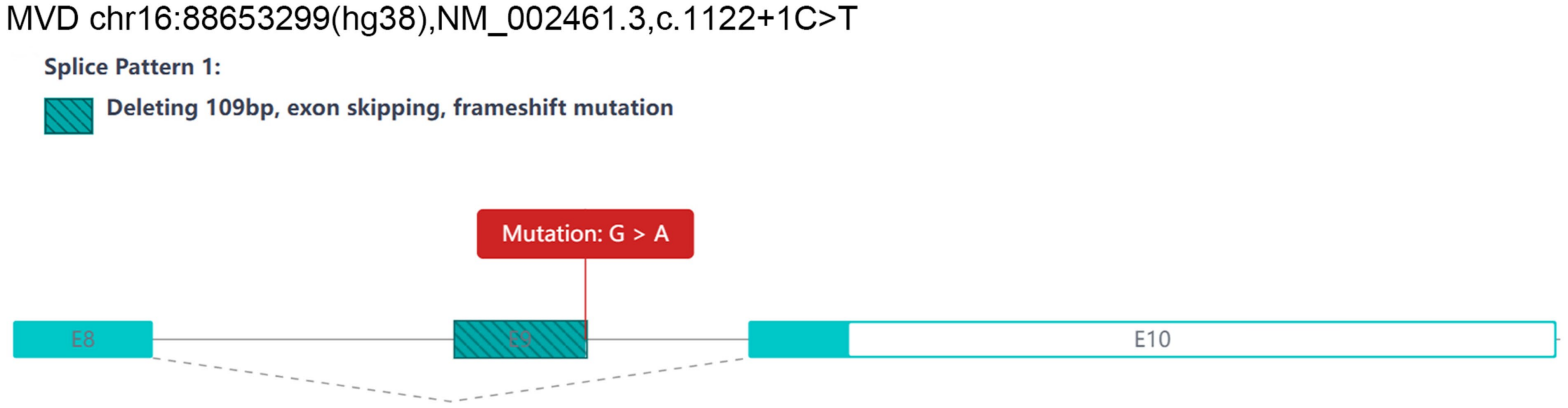
Prediction results of RDDC RNA splicer algorithms: the splice site mutation *MVD* c.1122 + 1G > A may lead to a change in the reading frame, exon skipping of exon 9, and deletion of a 109 bp fragment, causing a frameshift mutation.

To assess the potential pathogenicity, four online tools were utilized: PROVEAN^3^ (10), PolyPhen-2^4^ (11), SIFT^5^ (12), and Mutation Taster^6^ (13). The novel *FDPS* mutation c.986A > C was classified as “deleterious” with a score of −4.78 by PROVEAN, “probably damaging” with a score of 1.000 by PolyPhen2, “Damaging” with a score of 0.00 by SIFT, and labeled “disease-causing” with a probability of 0.999 by Mutation Taster. Similarly, the other novel *MVD* mutation c.576G > T was characterized as “deleterious” with a score of −12.78 by PROVEAN, “probably damaging” with a score of 1.000 by PolyPhen2, “Damaging” with a score of 0.00 by SIFT, and recognized as “disease-causing” with a probability of 0.999 by Mutation Taster. Furthermore, the splice site mutation c.1122 + 1G > A was analyzed using the RNA splicer algorithms provided by the Rare Disease Data Center (RDDC). The results suggest that this specific mutation may lead to a change in the reading frame, skipping of exon 9, and deletion of a 109 bp fragment, resulting in a frameshift mutation (Figure 5).

### 3.4 Review of *MVD* mutation c.746 T > C in Jiangxi Province

In our earlier research, Wang et al. reported six patients with *MVD* mutations, three of whom had the c.746 T > C mutation (8). In conjunction with this study, we identified a total of six patients with the *MVD* c.746 T > C mutation from Jiangxi province, China, representing 50% of the total *MVD* mutation cases (12 in total). These patients are all sporadic cases, comprnsisting of four males and two females, with an age of onset ranging from 3 to 67 years. All these individuals hail from the northern part of Jiangxi Province in China, and there is no evidence of familial connections among them in recent generational lines. They include two cases of DSAP, three cases of DSP, and one case of LP.

## 4 Discussion

In Zhang et al. (14) conducted a study utilizing exome sequencing on individuals diagnosed with DSAP, which led to the identification of mutations in the *MVK* gene. In 2015, it was confirmed that mutations in genes encoding three other mevalonate pathway genes (*MVD, FDPS*, and *PMVK*) also contributed to the occurrence of DSAP and various other PK subtypes (3, 15, 16). In our study, we identified two novel *MVD* mutations and one novel *FDPS* mutation in cases of PK from Jiangxi province, China.

The *MVD* gene, situated in the 16q24.1–24.3 locus, comprises 10 exons and encodes Mevalonate 5-diphosphate decarboxylase. This enzyme facilitates the decarboxylation of mevalonate 5-diphosphate in an ATP-dependent manner, resulting in the production of isopentenyl diphosphate, which is a vital precursor for dolichol, cholesterol, ubiquinones, isoprenylated protein, and sheme A. In our study, the novel mutation *MVD* c.1122 + 1G > A was found in sporadic case 2. According to predictions made by the RNA Splicer tool, this particular mutation disrupts the normal splicing process by leading to the exclusion of exon 9. The skipping of this exon results in a frameshift mutation, which in turn triggers alternative splicing and consequently has a significant impact on the coding sequence of the protein. Hence, we hypothesize that this mutated protein is probably nonfunctional, contributing to disease pathogenesis. Additionally, the *MVD* mutation c.576G > T, observed in sporadic case 7, is categorized as a missense mutation. The protein structure prediction model suggests that the p. GLN329PRO mutation replaces GLN329 with PRO, which removes the hydrogen bonds with GLY325 and THR326. We speculate that these alterations induce conformational changes and functional impairments in the protein, leading to the onset of PK.

The *FDPS* gene, found at chromosome 1q22, consists of 10 exons and encodes the enzyme Farnesyl diphosphate synthase (17). This enzyme catalyzes the conversion of isopentenyl pyrophosphate and dimethylallyl pyrophosphate into geranyl pyrophosphate and farnesyl pyrophosphate (18). Farnesyl diphosphate, the ultimate product of this enzymatic process, is essential for cholesterol synthesis and acts as a ligand or agonist in interactions with specific hormones and proteins. In our study, a novel *FDPS* mutation, c.986A > C, was found in sporadic case 4. Protein structure modeling indicates that in the p. Trp192Cys mutant protein, the side chain of Cys192 engages in weak alkyl interactions with Arg247 and Leu195, resulting in the complete abrogation of the hydrogen bond formed by wild-type Trp192, leading to functional loss of the protein. To date, only 10 mutations (c.1129_141 + 994del, c.283–1776_649–143del, c.200 T > C, c.338G > A, c.438 T > G, c.486 + 1G > A, c.535C > T, c.634_635delTA, c.773 + 1G > A, and c.1163_1164insA) in the *FDPS* gene have been reported in patients with PK (3, 8, 19–22). It has been noted that the skin lesions present in PK patients with *FDPS* mutations display a homogeneous and superficial nature (19). The lesions observed in sporadic case 4 are consistent with this characteristic.

The mutation *MVD* c.746 T > C has been reported as a hot-spot mutation (3). In our study, the *MVD* mutation c.746 T > C was observed in sporadic cases 1, 3, and 7. In conjunction with our previous study, we identified a total of six patients with the *MVD* c.746 T > C mutation from Jiangxi province, China, representing 50% of the total *MVD* mutation cases. Similarly, Leng et al. observed that this mutation was found in three families, constituting 50% all patients with *MVD* mutations in their cohort (23). Furthermore, despite carrying the same mutation, the age of onset and clinical subtype varied among patients. This variability may be associated with the development of PK, influenced by factors such as ultraviolet exposure, genetic susceptibility, immunosuppression, radiation, medications, and viral infections.

Genetic insights into the pathogenesis of PK provide guidance for therapies directed at specific pathophysiological mechanisms. The mevalonate pathway produces various end products that are critical for numerous cellular functions, including cholesterol, geranylgeranyl pyrophosphate, ubiquinone, dolichol, isopentenyladenine, and farnesyl pyrophosphate. Dysregulation of the mevalonate pathway decreases the production of these end products, leading to the accumulation of toxic metabolites. Future therapeutic directions should focus on correcting the metabolic anomalies resulting from the disordered mevalonate pathway. A novel topical treatment targeting the HMG-CoA reductase enzyme, utilizing 2% lovastatin, along with the replacement of cholesterol as an end product of the cholesterol synthesis pathway (2% cholesterol), has been described in the treatment of PK (24). A recent study indicated that both lovastatin 2% plus cholesterol 2% and lovastatin 2% alone equally improved lesions of DSAP over the study period, suggesting that cholesterol may not be necessary in formulations and that lovastatin cream may serve as a new primary treatment option for patients diagnosed with DSAP (25). However, there is currently a lack of therapeutic studies targeting other end products of the mevalonate pathway. Therefore, further research in this area will help provide new therapeutic directions for the treatment of PK.

This study has several limitations. Firstly, the analysis of the pathogenic mechanisms of novel gene variants primarily relies on bioinformatics analyses. Such bioinformatics-based predictive analyses can only infer potential pathogenic mechanisms based on the impact of a variant on protein structure. Therefore, functional experiments for further validation are necessary. Additionally, the number of patients analyzed was relatively small.

## 5 Conclusion

In conclusion, we identified two novel *MVD* mutations (c.1122 + 1G > A, c.576G > T), and one novel *FDPS* mutation (c.986A > C). This research has expanded the database of mevalonate pathway genes associated with PK, thereby improving our comprehension of the fundamental mechanisms involved. Further studies will focus on functional studies to elucidate the molecular mechanisms underlying PK pathogenesis.

## Data Availability

The datasets presented in this study can be found in online repositories. The names of the repository and accession number(s) can be found below: NCBI GenBank; PX769337, PX769338 and PX769339.

## Footnotes

1
https://genome.ucsc.edu

2
http://zhanglab.ccmb.med.umich.edu/I-TASSER/

3
http://provean.jcvi.org/index.php

4
http://genetics.bwh.harvard.edu/pph2

5
http://sift.bii.astar.edu.sg

6
http://www.mutationtaster.org
