# Correction: Identification of two novel *MVD* mutations and one novel *FDPS* mutation in Chinese patients with porokeratosis

**DOI:** 10.3389/fmed.2026.1799246

**Published:** 2026-02-09

**Authors:** Frontiers Production Office

**Affiliations:** Frontiers Media SA, Lausanne, Switzerland

**Keywords:** Chinese, *FDPS*, mutation analysis, *MVD*, porokeratosis

For authors Liang Wu and Chunming Li, the equal contribution footnote was incorrectly published as:

“^†^These authors have contributed equally to this work and share first authorship”

The correct equal contribution footnote appears below.

“^†^These authors have contributed equally to this work”

The original version of this article has been updated.

